# Simultaneous structures in sign languages: Acquisition and emergence

**DOI:** 10.3389/fpsyg.2022.992589

**Published:** 2022-12-22

**Authors:** Cornelia Loos, Austin German, Richard P. Meier

**Affiliations:** ^1^Institute of German Sign Language and Communication of the Deaf, Universität Hamburg, Hamburg, Germany; ^2^Department of Linguistics, University of Texas at Austin, Austin, TX, United States

**Keywords:** simultaneity, classifier constructions, language emergence, language acquisition, discourse

## Abstract

The visual-gestural modality affords its users simultaneous movement of several independent articulators and thus lends itself to simultaneous encoding of information. Much research has focused on the fact that sign languages coordinate two manual articulators in addition to a range of non-manual articulators to present different types of linguistic information simultaneously, from phonological contrasts to inflection, spatial relations, and information structure. Children and adults acquiring a signed language arguably thus need to comprehend and produce simultaneous structures to a greater extent than individuals acquiring a spoken language. In this paper, we discuss the simultaneous encoding that is found in emerging and established sign languages; we also discuss places where sign languages are unexpectedly sequential. We explore potential constraints on simultaneity in cognition and motor coordination that might impact the acquisition and use of simultaneous structures.

## Introduction

Signed and spoken languages differ typologically in a key aspect of their structure. Spoken languages are largely organized sequentially (Pinker and Bloom, 1990), both in their phonology (strings of phonemes) and in their morphology (a tendency toward prefixation and suffixation). Signed languages show much more simultaneous structuring, whether in their phonology or morphology (Klima and Bellugi, 1979; Meier, 2002; Aronoff et al., 2005). This difference is not absolute. There are simultaneously organized structures in spoken languages as well: notably the tonal morphology of many African languages (Odden, 1995), as well as some Mesoamerican languages such as Chatino (Cruz, 2011) and Rarámuri (Caballero and German, 2021). For instance, Rarámuri *tisô* ‘walk with a cane (bare stem)’ and *tisò* ‘walk with a cane (imperative singular)’ are distinguished by falling tone vs. low tone, respectively (Caballero and German, 2021: 160). Likewise, there are sequentially organized constructions in signed languages, most obviously in their syntax and in compounding, but also instances of prefixation and suffixation that have been reported in American and Israeli Sign Languages (Aronoff et al., 2005).

Overall, however, children and adults who acquire a sign language need to learn, produce, and comprehend more simultaneous structures than individuals acquiring a spoken language. Simultaneity can be seen as an outcome of the constraints of the manual articulators (for example, the slow rate of signing—Klima and Bellugi, 1979), of the availability of multiple articulators to signed languages, of the capacities of the visual system, and/or of the resources for iconic representation that the visual-gestural modality affords; see Meier (2002) for discussion. The bandwidth available to the human visual system apparently means that such layered information can be apprehended successfully (Meier, 1993). Here we investigate what challenges simultaneity may pose for the grammars of signed languages, for the children acquiring signed languages as first languages, for the adults learning them as second languages, and for the emergence of new signed languages.

Articulated and perceived in the visual-gestural modality, signed languages have multiple articulators that can move independently or semi-independently at the same time. The hands, arms, torso, head, and various facial muscles may encode different types of linguistic information simultaneously, from phonological contrasts to spatial relations and information structure (Aronoff et al., 2005; Sandler and Lillo-Martin, 2006; Vermeerbergen et al., 2007). Vermeerbergen et al. (2007) distinguish three types of simultaneity: manual simultaneity, manual-oral simultaneity, and the simultaneous use of several non-manual articulators or of a manual and a non-manual articulator other than the mouth. In this paper, we are mostly concerned with manual simultaneity, where each hand contributes meaning. Building on the work of Miller (1994), we can distinguish the following five subtypes of manual simultaneity according to the types of signs combined and the temporal coordination between the hands: (a) two lexical signs are produced simultaneously, (b) two classifiers are produced at the same time, (c) one hand produces a sign and then holds it while the other hand continues signing one or more signs (weak-hand hold), (d) the non-dominant hand produces an enumeration morpheme while the dominant hand encodes the items on the list, and (e) one hand produces an index sign (or “pointer buoy,” Liddell, 2003) while the other produces a string of signs. Sign languages may differ in the extent to which they use simultaneous encoding; for instance, Nyst (2007) reports that Adamorobe Sign Language exhibits little manual simultaneity.

Manual-oral simultaneity involves synchronized productions of the hands and mouth (either via mouthings or mouth gestures), which may contribute the same or complementary information. In German Sign Language (DGS), for example, one might sign GUT ‘good’ while mouthing the equivalent German word, and both contribute the same information. One may also sign GUT while mouthing *alles* ‘all’, where the mouthing contributes an argument of the predicate GUT. More generally, other non-manuals may be combined simultaneously, e.g., raised eyebrows and a headshake in negative polar questions, and they may (further) combine with manual signs. While we focus on manual simultaneity in this paper, we will sometimes draw on manual/non-manual simultaneity when discussing constructed action and the acquisition of simultaneous structure in discourse.

The availability of multiple articulators is not necessary for simultaneous structure in signed languages. Even signs that are produced by just one hand may show simultaneous structure. For example, one-handed “classifier constructions” (CCs)^1^ express properties of the referent through the handshape (whether it is a human, or a vehicle, or a small animal), while the location and movement of the sign simultaneously encode the location and/or the movement direction of that referent, as well as additional information about, for instance, its manner of movement. Likewise, inflectional and derivational morphology in signed languages is typically simultaneous in its structure. The distinction between one- and two-handedness is a feature of some inflectional categories [e.g., certain dual verb forms in American Sign Language (ASL) and some plural nouns in Sign Language of the Netherlands (NGT), van Boven, 2021] and some derivational categories (e.g., the characteristic adjectives of ASL, Klima and Bellugi, 1979; Padden and Perlmutter, 1987). However, inflection and derivation are largely signaled by changes in movement patterning that affect the overall movement contour of a sign and that are independent of the handedness of signs. These modulations of movement structure are non-affixal; examples include the changes in movement direction and in hand orientation by which directional verbs in many signed languages mark argument structure (e.g., Lillo-Martin and Meier, 2011). Other examples include the short, repeated, restrained movement that marks deverbal nouns in ASL (Supalla and Newport, 1978; Abner, 2019) and the varying patterns of repeated movement that mark temporal aspect in ASL (Klima and Bellugi, 1979).

In this paper, however, we focus on the simultaneous linguistic structure that arises from the availability of two semi-independent manual articulators in the visual-gestural modality. We begin by discussing children’s acquisition of two-handed signs and of the motoric factors that may affect the production of those signs. We then turn to the development of the use of the non-dominant hand in discourse. Lastly, we address children’s use of the two hands in CCs to describe the Figure and Ground of a motion event. In our discussion of classifier constructions, we compare children and adult learners’ acquisition of Figure and Ground to their acquisition of one-handed Path and Manner constructions. This comparison will give us insight into the role that two-handedness plays in the linguistic and developmental constraints affecting classifier constructions. Throughout the discussion we will present findings from both first and second language acquisition and will also bring in relevant data from the emergence of new signed languages.

## Simultaneity in the lexicon: The two hands

A fundamental resource for languages in the visual-gestural modality is the two hands that the human body makes available. Spoken languages have no counterpart to these paired articulators. However, the two hands are only partially independent. There are developmental and linguistic constraints on the simultaneous action of the two hands, and thus there are limits to how much information they can encode simultaneously.

### Two-handed signs in the lexicons of signed languages

**FIGURE 1:**
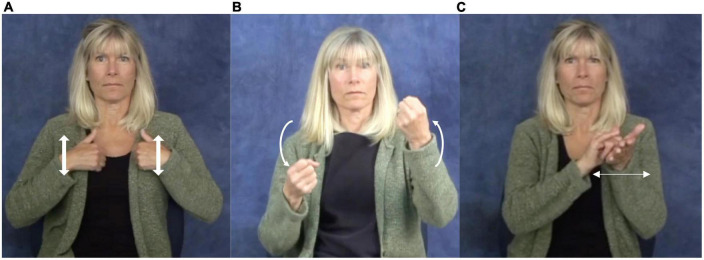
The American Sign Language (ASL) signs **(A)** BATH, **(B)** CAR, and **(C)** NEW-YORK. Reproduced with permission from Prof. Naomi Caselli, available at https://asl-lex.org/.

In natural signed languages, there are three values for the “hand arrangement” parameter (Klima and Bellugi, 1979). Signs may be one-handed or two-handed; among two-handed signs, the non-dominant hand may move or may be held in place. As has long been observed, the natural signed languages reported to date constrain the form of two-handed signs (Battison, 1978; Eccarius and Brentari, 2007). Two-handed “symmetrical” signs are ones in which both hands move; the two hands must show the same movement, whether in phase (e.g., the ASL sign BATH in Figure 1A)^2^ or out of phase (e.g., the ASL sign CAR in Figure 1B); they must also share the same general location^3^ and handshape [thereby barring artificial signs such as TOTAL-COMMUNICATION, which has a T (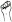) handshape on the non-dominant hand and a C (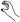) on the dominant].^4^ Signs falling within the second class of two-handed signs show a static non-dominant hand, sometimes called a “base” hand; see the ASL sign NEW-YORK in Figure 1C. These signs may have distinct handshapes on the dominant and non-dominant hands, but the non-dominant is only permitted a limited number of relatively basic handshapes. These constraints on sign formation seem related to issues in bimanual coordination; they limit the motoric complexity of monomorphemic signs. These constraints also have the effect of reducing the set of possible phonological contrasts in that no two-handed, symmetrically moving sign may have distinct handshapes on the two hands. These constraints can thus also be seen as limitations on the linguistic complexity of lexical signs (but see Eccarius and Brentari, 2007, for an application of these constraints to CCs).

### Developmental issues in bimanual coordination

Separate control of the two hands during object manipulation emerges late in the first year of life; for example, Fagard et al. (1994) reported considerable development between 6 and 12 months in infants’ abilities to coordinate the use of their two hands to perform means-ends tasks that require one hand to hold a box open while the other hand retrieves a toy. Younger infants showed better performance in tasks that could be performed sequentially, rather than tasks requiring the participation of both hands simultaneously.

To perform one-handed movements, children must be able to inhibit the action of the inactive hand. However, when one-handed action is planned, the child’s other hand may sometimes mirror that action. This can persist into adolescence for some movements. For example, Connolly and Stratton (1968) reported that, at age five, roughly 55% of boys and 30% of girls showed mirror movements of the non-dominant hand when asked to raise just the middle finger of their dominant hand while their palms were resting flat on a table; by ages eight to nine more than 80% of all children successfully inhibited the non-dominant hand. But at ages 12 to 13 most children still showed mirror movements on a finger-spreading task. Wolff et al. (1983) tested typically developing, right-handed 5- and 6-year-olds three times over 12 months; in general, mirror movements declined over this period. For example, there was a significant decline in the number of 5-year-olds who produced mirror movements in a task in which they were asked to repeatedly pronate and supinate one hand.

Toddlers who were observed longitudinally in a bimanual drumming task did not show stable out-of-phase coordination of the two hands until 20 months (Brakke and Pacheco, 2019, who use the term “anti-phase”); signs such as ASL CAR (Figure 1B) show this out-of-phase relationship in that one hand moves down while the other moves up. Some aspects of bimanual coordination (e.g., timing) do not mature until ages nine to eleven, as probed by bimanual finger-tapping tasks (Wolff et al., 1998). Mature bimanual coordination may require functional maturation of the corpus callosum, which has been thought to occur at age ten to eleven (Yakovlev and Lecours, 1967). Transference of information between the two hemispheres through the corpus callosum may enable the inhibition of unintended mirror movements by the hand that is not being intentionally moved by the child (Geffen et al., 1994).

### Acquisition of two-handed signs by deaf children

Relevant data on how bimanual coordination may affect the acquisition of signs is less rich than we would wish. Siedlecki and Bonvillian (1993) report a diary study of the early acquisition of ASL vocabulary by nine children (ages 5−18 months) of deaf parents; eight of the children were hearing and one was deaf. During visits to the children’s homes, parents were asked to demonstrate on videotape how their children had produced the signs that the parents had identified in their diaries; deletion of a stationary non-dominant hand was observed, but infrequently (5/62 signs). The parents identified just two errors in which the non-dominant hand moved symmetrically with the dominant hand. Deletion of the non-dominant hand from symmetrical two-handed signs was significantly more frequent (29/135 target signs). Interestingly, Siedlecki and Bonvillian (1993) interpret their data to suggest that children’s errors were constrained by whether distinctive phonological information would be lost.

Cheek et al. (2001) examined the prelinguistic gesture (including communicative gestures and “manual babbles”) of ten children, five sign-naïve hearing infants and five deaf infants born to deaf, ASL-signing parents. Gestures with a static non-dominant hand were essentially absent from their data; just two tokens were identified from the deaf infants and none from the hearing infants. These authors also examined videotaped, naturalistic data on the production of ASL signs by four native-signing deaf children. Those children were followed longitudinally from as early as 5 months to as late as 17 months. Across this age span, the vast majority of one-handed signs (411/442 tokens, 93%) and most two-handed symmetrical signs (83/117 tokens, 71%) were produced correctly with respect to the hand arrangement parameter. Errors on two-handed symmetrical target signs dropped the non-dominant hand, which can be grammatical in the adult language. The relatively few errors on one-handed target signs involved the addition of a symmetrically moving non-dominant hand. This last type of error might be viewed as consistent with children’s mirroring behavior on non-linguistic tasks. Shield et al. (2020) observed the fingerspelling of a native-signing hearing child of deaf parents who has autism spectrum disorder. At 10;2, this boy’s non-dominant arm mirrored the large proximal movements of his arm associated with his production of ASL’s one-handed fingerspelling system; he does not seem to have mirrored the handshapes themselves. At 14;11, these mirror movements were absent. One question for future research is whether such mirror movements are restricted to motorically and perhaps cognitively demanding signing such as fingerspelling.

Base-hand signs appear to be poorly represented in Cheek et al.’s (2001) data vis-à-vis their representation in the lexicon of ASL; there were just 62 tokens out of a total sample of 629 sign tokens. In contrast, 25% of the entries in Stokoe et al.’s (1965) *Dictionary of ASL on Linguistic Principles* have a non-dominant base hand (Klima and Bellugi, 1979), as do 25% of signs listed in the ASL-LEX lexical database (Caselli et al., 2017; Sehyr et al., 2021). An inspection of the ASL adaptation of the MacArthur Communicative Development Inventory revealed that just two of the 35 earliest-produced signs have a static non-dominant hand; those signs were TREE and COOKIE (Anderson and Reilly, 2002).

As noted, Cheek et al. (2001) only identified 62 tokens with a base hand in the target sign. Error rates on this class of signs were higher than on one-handed signs or symmetrical two-handed signs. To correctly produce adult target signs that have a static non-dominant hand, children must inhibit movement of that hand. Cheek et al.’s subjects were successful on 30 tokens. Of the 32 errors, 12 simply dropped the non-dominant hand. However, in 20 tokens, the two hands moved symmetrically; for example, when one child (age 1;4.6) produced the sign FALL, both hands moved downward in tandem. In contrast, the adult target involves a downward movement of the dominant hand to a static non-dominant hand. Lastly Marentette and Mayberry (2000) reported a case study of one native-signing deaf girl’s acquisition of ASL from 12 to 25 months. They briefly describe two relevant classes of errors: (1) errors in which the child froze the movement of the non-dominant hand in signs that have symmetrical movement of the two hands in the adult target (e.g., SHOE, BOOK), and (2) errors in which the non-dominant hand mirrored the movement of the dominant hand (COOKIE, SCHOOL). Productions of these error types peaked at 23 months.

Instances have been reported in which alternating movement of the two hands - that is, movements in which the two hands execute the same movement, but out of phase - was replaced by movements in which the two hands moved in phase (Newport and Meier, 1985). Szameitat (2009) examined the acquisition of ASL phonology by twelve 24-month-old deaf children; six of these children showed at least one instance of “synchronization”, by which the two hands moved in phase rather than out of phase. For two children, synchronization was frequent in their sign productions.

In sum, our review finds limited published data that would allow us to assess the impact of motor control issues in bimanual coordination on children’s early sign production. What data we do have suggests that children are broadly successful in producing the correct hand arrangement of adult target signs. Evidence on the acquisition of signs with a static non-dominant hand is scant, in part because children seem to attempt few such signs. Here the naturalistic video data reported by Cheek et al. (2001) provides limited evidence that children sometimes err by failing to inhibit movement of the non-dominant hand. The Shield et al. (2020) report raises the possibility that some atypically developing children may have lingering problems in inhibiting the non-dominant hand even in the production of one-handed signs. Very clearly, we need more data - especially perhaps from older children - that would address the question of whether children’s production of two-handed signs is constrained by motor control issues.

### The use of the two hands outside the lexicon

**FIGURE 2:**
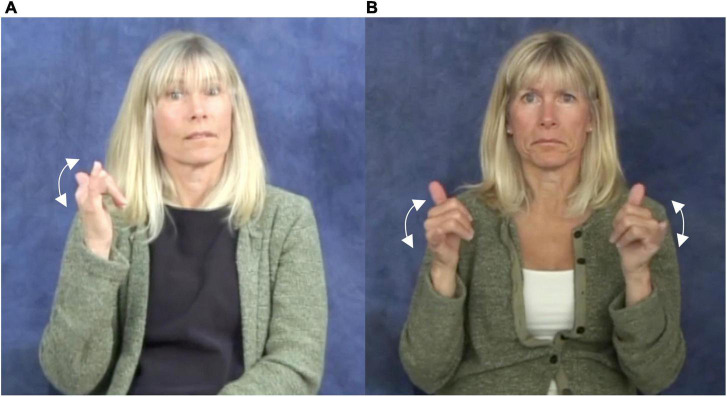
The ASL signs **(A)** PLAY and **(B)** YELLOW. Reproduced with permission from Prof. Naomi Caselli, available at https://asl-lex.org/.

In the lexicon of ASL, the non-dominant hand in symmetrical signs is generally redundant. There are few minimal pairs that differ just in whether two signs have one vs. two moving hands; examples noted in the literature include ASL YELLOW/PLAY (Figure 2; see Klima and Bellugi, 1979) and DEAD/PERSON(AL) in Swedish Sign Language (Börstell et al., 2016).

Outside the lexicon, however, the non-dominant hand encodes important information in a variety of simultaneously organized constructions. Adding a second hand to a one-handed monomorphemic sign can be morphologically significant; in ASL, the doubling of the two hands is a feature of the marking of the dual and reciprocal forms of some one-handed verbs (e.g., GIVE), of certain distributive plurals (Klima and Bellugi, 1979), and of the characteristic adjective form of adjectival predicates referring to temporary or incidental states (Klima and Bellugi, 1979; Padden and Perlmutter, 1987). Doubling of the hands, in combination with alternating movement, can mark the plurals of some nouns in various signed languages (Pfau and Steinbach, 2006).

The non-dominant hand plays a crucial role in CCs and may also assume important functions in discourse regulation. It is these constructions to which we now turn. Here we might expect motoric complexity to be a limiting factor for the young child. We first turn to the acquisition of discourse functions of the non-dominant hand and then discuss Figure-Ground constructions, where the static non-dominant hand encodes information about the landmarks against which objects move.

## Acquisition of narrative and discourse functions of simultaneity

Some of the earliest studies on manual simultaneity mention its discourse-pragmatic functions. Engberg-Pedersen (1994) and Miller (1994) look respectively at DTS (*Dansk tegnsprog*) and LSQ (*Langue des signes québécoise*). They claim that one of the main functions of manual simultaneity is to distinguish foregrounded from backgrounded information such that the dominant hand typically carries information that is central to an ongoing discourse. The non-dominant hand may modify this information or otherwise contribute to the “management of the discourse situation” (Miller, 1994: 103). It may, for instance, maintain a topic referent via a weak-hand hold as illustrated in (1a) (Friedman, 1975; Gee and Shepard-Kegl, 1983), or indicate the spatial or temporal frame of a described event (1b).

**Table:** 

(1)	a.	R: WE LOOK-AT IX_car_ WE LOOK-AT IX_car_
		L: CAR - - - - - - - - - - - - - - - - - - - - - - -
		‘We looked at the car. We looked at the car.’

	b.	R: ENGLISH CLASS GO HOME STUDY
		L: TWO (o’clock)- - - - FOUR SIX- - - - - -
		R: EAT
		L: SEVEN
		‘At two (I go to) English class; from four to six (I) go home and study; at seven (I) eat.’ (Friedman, 1975: 953)

Few studies to date have focused on the acquisition of discourse structure in signed languages and yet fewer have discussed discourse-structural uses of bimanual simultaneity in child language development. Prinz and Prinz (1985) report that children acquiring ASL start using weak-hand holds for topic maintenance and topic chaining around age ten. Younger signers (age 8−9) may briefly display a sign on the non-dominant hand, but then drop the hand despite using the same sign at a later point within the same discourse episode, indicating that its referent was a topic in the child’s narrative. Tang et al. (2007) observe that learners of Hong Kong Sign Language (HKSL), even as late as age 13, rarely used such discourse-structuring weak-hand holds (“fragment buoys” in Liddell, 2003). The late emergence of weak-hand holds for topic maintenance parallels the emergence of other discourse-structuring devices such as backchanneling head nods or lexical signs of agreement (e.g., OKAY, SAME) around the same age (Prinz and Prinz, 1985). Prinz and Prinz concluded that the strategies employed by deaf children converge with the development of similar skills in spoken languages, e.g., how discourse topics are initiated, maintained, and terminated. Although it is possible that the late emergence of discourse weak-hand holds in sign languages is conditioned in part by persistent motor coordination difficulties in articulating a static base hand, the timeline by which these usages are acquired in signed languages does not seem at variance with the timeline by which narrative skills are acquired in spoken languages (Clark, 2016) or by which other discourse-structuring devices are acquired in signed languages.

The nascent use of both hands for creating topic-comment structures is also observed in homesigners around the same age. Scroggs (1981) describes the productions of a 9-year-old deaf boy, Alexander, who at the time of recording had had little exposure either to ASL or signed English from his hearing parents, and who had just been enrolled in a public day-school program for deaf children in which a form of signed English was used. His narrative productions frequently contained a topic established on the dominant hand that was then moved to the non-dominant hand while the dominant hand articulated a description or comment. In one example, Alexander described the speed of a motorcycle by first producing the motorcycle on his right hand, then moving it to the left hand while producing an idiosyncratic sign for ‘speed’ on the right hand. Another example was Alexander’s description of a surfer rescued by a helicopter, in which he represented the discourse topic ‘surfer’ on his left hand and the helicopter whirling to the rescue on the right hand.

Even without the additional challenges of bimanual coordination, young children struggle to encode concurrently unfolding events. Reporting such events poses cognitive and linguistic challenges to children acquiring English, as evidenced by the fact that connectives such as *while* appear after markers of temporal sequence such as *then* or *next* and are not used productively until after age seven (Morgan, 2002). These challenges are attributed to the demands of having to keep the actions of more than one character in an event in mind, so younger children tend to focus on a single main character instead.

Adult signers may combine constructed action (CA) with lexical signs or CCs to represent the concurrent actions of more than one character. An adult BSL signer who retold the Frog Story described a boy falling from a tree while an owl emerged from it via the simultaneous production of a whole entity classifier for the boy and CA to represent the owl (Morgan, 2002). Children aged four to six exhibited no such combinations of CA and CCs. Children aged seven to ten still presented concurrent events sequentially by focusing on one character at a time, but their signing spaces started showing overlap. Older children aged 11−13 used sequential strategies like sandwiching one event between two mentions of another event. For instance, the boy in the Frog Story falls from a tree while his dog is being chased by bees; one child signed the boy’s fall followed by the dog being chased, and then depicted the boy falling again. The children also used lexical means such as a verb of perception to encode temporal concurrence (e.g., SEE in “the dog sees the boy fall from a tree”). Importantly, none of the children in Morgan’s study were reported to represent two characters’ actions simultaneously by combining CA and classifier or lexical predicates.

Recent studies on the acquisition of a signed language by adult users of a spoken language (M2L2 learners, or “second modality, second language learners”) show that adult L2 learners behave in similar ways to child L1 learners when it comes to the expression of simultaneous structure. Gulamani et al. (2022) looked at re-tellings of the Frog Story by 23 intermediate learners of BSL and noted that their use of CA was less frequent than CCs, one of the reasons being that it requires the coordinated use of more articulators. Gulamani et al. (2022) considered the articulations of the dominant hand, the non-dominant hand, the body, eyebrows, eyes, mouth, and head. The adult native participants in their study used five to seven articulators simultaneously substantially more frequently than the M2L2 learners, who used one to three articulators more frequently than the native signers. The authors suggest that the comparatively low information density in M2L2 narratives as compared to L1 narratives is due to the cognitive difficulties of (a) coordinating the articulation of several articulators and (b) keeping in working memory all relevant aspects of a scene while accessing a still developing language system.

## Acquisition of classifier constructions

One class of simultaneous expressions in sign languages that are enabled in part by the availability of two manual articulators are CCs. CCs form a system of schematic visual representations that are attested in most signed languages; they differ from lexical signs in that each of their formational components bears meaning. Importantly, the two hands may encode morpho-syntactically independent predicates (Zwitserlood, 2003) that in most accounts consist of a semantically light movement root and a classifier handshape (e.g., Benedicto and Brentari, 2004). Classifier handshapes in signed languages are morphemes denoting the semantic class, size, or form of the entity whose movement or location is being described, for example, a vehicle, airplane, small animal, or human (Supalla, 1982). CCs primarily denote spatial relations and the movement of entities, such that the handshape of each hand represents an entity involved in the event, the place of articulation in the sign space represents the location of an entity or the relative spatial orientation of entities with respect to each other, and the movements of the hands show the path and manner of motion of those entities (Zwitserlood, 2012).^5^

Classifiers and the constructions containing them are only mastered around age eight (Supalla, 1982; Schick, 1990a; Slobin et al., 2003).^6^ This may be due to a number of independent properties of these constructions. First, representing more than one event participant simultaneously depends on the ability to use the two hands independently and may, as we have seen in Section “Acquisition of two-handed signs by deaf children,” be motorically demanding. Second, these constructions allow the encoding of many event components simultaneously and may therefore place high cognitive demands on the child. We will first discuss the simultaneous encoding of Figure and Ground in bimanual CCs, which illustrates both of the above challenges, and then turn to the simultaneous expression of Manner and Path of movement as an example of the cognitive challenges of encoding multiple event components even in one-handed signs.

### Figure and Ground

One property of events that is often expressed simultaneously in CCs is the involvement of more than one entity. In locative expressions, this typically involves a Figure that either moves, or is located, with respect to a Ground entity. Figures are typically smaller and foregrounded while Grounds represent larger, backgrounded entities. According to Özyürek et al. (2010: 1120), the canonical structure of locative expressions across signed languages first introduces the Ground (Gr) via a lexical sign; that sign is followed by a CC that locates the Ground in the signing space. The non-dominant hand (ND) holds the final position of this CC while the dominant hand (D) introduces the Figure (Fig) via a lexical sign followed by a CC showing either the location or movement of the Figure in relation to the Ground. This structure is represented in (2), and has been attested in ASL, DSL, DGS, LSQ, BSL, Taiwan SL, and HKSL (Özyürek et al., 2010).

**Table:** 

(2)	ND: [Gr NP] [Locate Gr] - - - - - -hold- - - - - -
	D: [Fig NP] [Locate Fig]

Supalla (1982) notes that Figure and Ground may be signed at the same time only if the signs representing them are one-handed. But if either the Figure or the Ground is represented by a two-handed sign, the motion verb encodes only the Figure, while the Ground is encoded by a preceding locative predicate. The grammatical possibilities for simultaneous expression are thus conditioned by the handedness of the constituent signs. It should be mentioned that, while the structure in (2) exhibits manual simultaneity in as much as Figure and Ground are signed (or at least held) at the same time, Özyürek et al. (2010) point out that simultaneity is not obligatory in encoding such locatives in Turkish Sign Language (TİD). They looked at descriptions of static spatial relations between two or more objects (e.g., boats on water, a painting on a wall), as well as motion descriptions of a Figure with respect to a Ground (e.g., a man walking toward a truck). Analyzing data on static spatial relations from six native TİD signers and on relative motion descriptions from four of the six signers, they found simultaneous Figure and Ground expression in just 1.4% of static spatial descriptions and in 20% of motion descriptions. Instead, signers often introduced and localized the Ground but did not hold it on the non-dominant hand when introducing the Figure, thereby requiring the addressee to keep the location of the Ground in mind. Using a similar study design, Perniss et al. (2015) also found a paucity of simultaneous Figure-Ground encoding in DGS, where only 7% of Figures were signed with respect to a Ground object held on the non-dominant hand. These findings raise questions about the frequency of simultaneous Figure-Ground constructions in the input to child learners of DGS and TİD, even those children who receive native input from deaf parents.

De Weerdt (2020) shows that simultaneous expression is influenced by whether Figure and Ground constitute new information or are already known to the interlocutors. Looking at production data from Finnish Sign Language, he notes that constructions with known Figures almost always triggered simultaneous descriptions (either of Figure and Ground or of Ground and a spatial adposition), while new Figures triggered simultaneous encoding in only 63% of descriptions. Perniss et al. (2015) claim that the simultaneous encoding of Figure and Ground marks non-default spatial relations between the two entities, for instance a boy standing on another boy’s shoulders. De Weerdt assumes that, given the higher cognitive load of encoding Figure and Ground simultaneously, this construction is more likely to occur when interlocutors are already familiar with both referents.

#### Acquisition of Figure and Ground

Children do not consistently include Ground information in their locative and motion CCs before age seven (Slobin et al., 2003 for ASL and NGT; Morgan et al., 2008 for BSL; Sümer, 2015 for TİD). Even in stative locative descriptions such as a ball sitting in a cup or a piece of paper lying under a bed, younger children will omit either Figure or Ground, with the Ground being omitted significantly more often (Sümer, 2015). Similar findings have been reported for HKSL (Tang et al., 2007), where learners were grouped by proficiency level rather than age or length of exposure.

Most studies report one common compensatory strategy: sequential predicates for Ground and Figure. An example given in Tang et al. (2007: 312) describes someone putting a hat on a bird’s nest. While adults would use the non-dominant hand to represent the bird’s nest by means of a located classifier, children first produced a one-handed sign to locate the bird’s nest, then signed an existential predicate for the hat, and lastly used a handling classifier to show the placement of the hat in the same location where the nest had previously been located. These sequential strategies further varied by whether the child linked the separate predicates via location or not. Younger children tended to set up new event spaces in signing space for each predicate and would, for instance, place the bird’s nest in a different location from the final (goal) location of the hat-moving predicate.

Children’s frequent omission of Ground elements does not seem to be due to an inability to form a conceptual representation of Ground. Even children with low HKSL proficiency in Tang et al.’s (2007) study sometimes used the non-dominant hand to express the Ground. Alternatively, the HKSL participants sometimes treated their own body as a Ground on which a Figure would move or be located. Tang et al. (2007) offered the following explanation for the absence of simultaneous Figure-Ground encoding in children’s productions: Descriptions of relative spatial location require the use of token space (Liddell, 1994). To use token space, children must abstract away from the signing space in front of them and project onto it an event space in which the articulators and the space itself stand in for something else; see Schick (1987), Morgan et al. (2008) for similar arguments based, respectively, on ASL and BSL acquisition data. In both ASL and HKSL, children make more errors with classifiers that use token space (entity classifiers) than with classifiers that use surrogate space (handling classifiers) (Tang et al., 2007; Morgan et al., 2008).

The cognitive load associated with this abstraction process may mean that something has to give elsewhere. A strategy for lowering cognitive load is to reduce the number of referents represented within one CC. Omitting the Ground appears to be the preferred means of achieving this. According to Sümer (2015), Ground objects are also less salient in dynamic motion events such as rolling a tomato up a hill, where the moving Figure draws attention away from the Ground.

#### Emergent signed languages

How does the acquisition of Figure and Ground encoding in established signed languages compare to the emergence of such encoding in young languages? Research on forms of gestural communication that, unlike conventional signed languages, have not been transmitted from generation to generation within a stable signing community provides the opportunity to probe the conditions under which simultaneous vs. sequential structures emerge. For instance, Goldin-Meadow et al. (1996) asked hearing non-signing adults to describe motion events using gesture, both with and without concurrent speech. They found that when participants produced gestures concurrently with speech, those gestures typically encoded information holistically, for instance using a gesture for a round object (the Figure) and moving it along some path. By contrast, when the participants were asked to produce gestures without speech, they produced a sequence of discrete gestures for each element of the motion event, e.g., using a gesture for a round object followed by a gesture tracing its path. Goldin-Meadow et al. (1996) argue that segmentation begins to arise when the full burden of communication is shifted to the manual modality.

Moving up the scale of conventionalization, we can also examine the kinds of structure that develop when a deaf child who cannot access spoken language and has not been exposed to a conventional signed language generates a novel sign system and continues to use it over an extended period of time as his or her primary means of communication, i.e., “homesign” (Goldin-Meadow and Feldman, 1975). Homesign systems represent an intermediate stage between gesture and full-blown signed languages. Despite the fact that these children have no systematic input from a conventional language, they nonetheless seek to communicate with their family members. We can examine the kinds of structures that develop when an isolated deaf child generates a novel sign system and continues to use it over an extended period of time as his or her primary means of communication.

According to Goldin-Meadow et al. (1995), American homesigners reliably produce sequences of discrete gestures for Figure and Path. Zheng and Goldin-Meadow (2002: 54) also observe that American and Chinese child homesigners “often produced separate gestures for the nominal elements of a motion event” (i.e., the Figure and Ground). The authors do not specify whether these gestures are ever produced simultaneously. Gentner et al. (2013) report that young Turkish homesigners (age range 3;8−5;6) rarely encoded Figure and Ground simultaneously. In the majority of their pertinent utterances (21/33), the children omitted one of these elements. Of the minority of utterances in which both elements were represented (12/33), only two contained simultaneous signs representing Figure and Ground. Morford (2002) elicited narratives from two adolescent homesigners; while both consistently represented Figure, neither explicitly represented Ground in any of their utterances.

**FIGURE 3:**
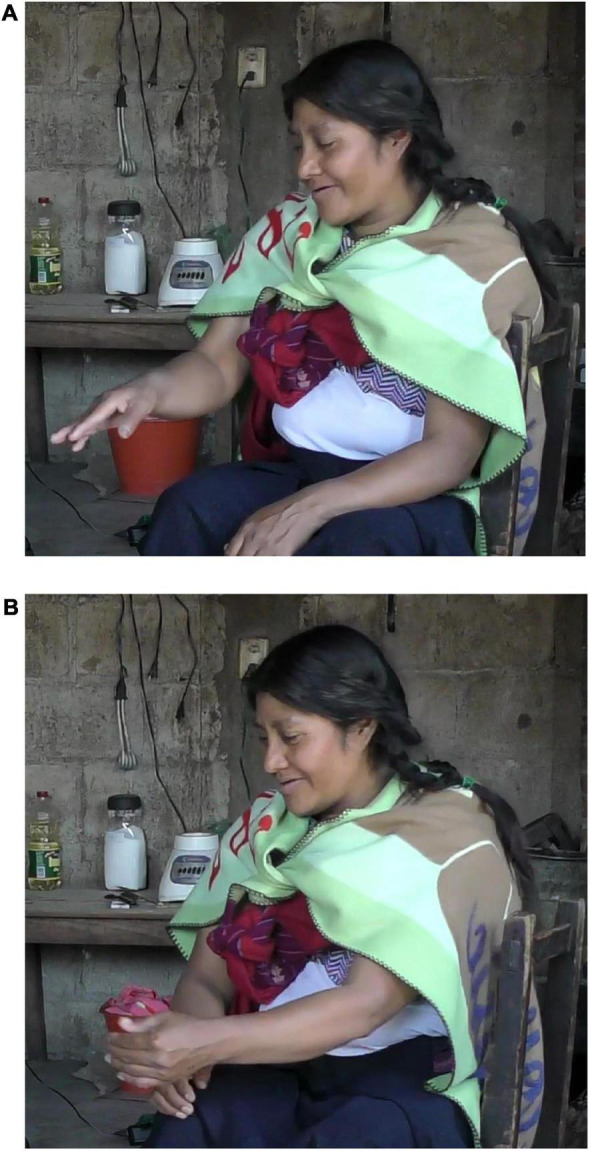
Sequential encoding of Figure and Ground in Zinacantec Family Homesign. **(A)** CC for Ground. **(B)** CC for Figure.

**FIGURE 4:**
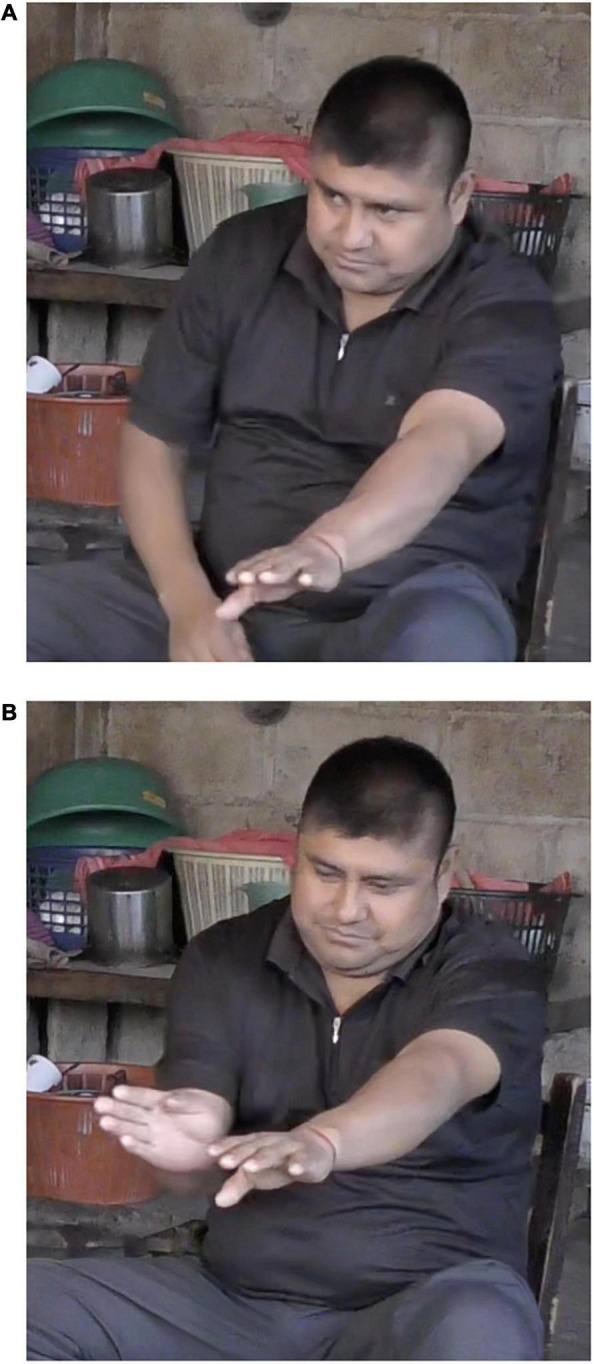
Simultaneous encoding of Figure and Ground in Zinacantec Family Homesign. **(A)** CC for Ground. **(B)** Simultaneous CCs for Figure and Ground.

Preliminary data from Zinacantec Family Homesign (ZFHS), an emergent sign language developed by three, now-adult, deaf siblings and their extended family members in southern Mexico, shed some light on how Figure and Ground are encoded in an emergent signed language (German, 2022a,b; see also Haviland, 2020). Descriptions of 40 motion events that included a moving Figure and a stationary Ground were elicited from all seven fluent signers of ZFHS (three deaf and four hearing). The eldest signer Jane typically encodes Figure and Ground with a sequence of separate CCs. For instance, in Figure 3 Jane describes a tricycle passing by a truck by first producing a CC for the Ground in the signing space in front of her, and then producing a second CC for the Figure, moving her hand past the location where the first CC was produced. By contrast, the later born ZFHS signers were generally more likely to encode Figure and Ground simultaneously. For instance, in Figure 4, the third deaf sibling Will describes the tricycle passing by the truck by first locating a CC for the truck in the signing space with his left hand. Then, using his right hand, he produces a CC for the truck by moving his right hand past his left hand, which maintains the CC for the hoop. The frequency of this simultaneous strategy increases as one moves from the oldest to the youngest signers.

#### M2L2 acquisition

Signers who first learn a signed language as (young) adults come to the table with more developed cognitive and motor coordination skills than child learners. Nonetheless, their error patterns, especially in the acquisition of CCs, exhibit similarities to those of child learners. Boers-Visker (2021) looked at two-handed CCs in 14 M2L2 learners of NGT and found that learners produced omission errors well into the second year of their studies. In contrast to L1 learners, however, they sometimes self-corrected their productions, adding in the Ground on the non-dominant hand while holding the dominant hand in place. This points toward a cognitive demand as the cause of the omission errors rather than a purely motoric difficulty. Most of the NGT learners (9 of 14) also sometimes resorted to sequential constructions in place of simultaneous ones, for instance when describing a car and a truck standing next to each other.

Studies on M2L2 learners of NGT and Norwegian Sign Language (NTS) find that learners have difficulties in coordinating the use of their hands in relation to each other, especially across longer stretches of discourse. Ferrara and Nilsson (2017) report that NTS learners (approximately 1.5 years of study) sometimes crossed their arms in depicting an entity’s movement, misjudging the hands’ distance from each other; they would place entities higher in signing space than others that were at the same height in real space. Boers-Visker (2021) noted that learners of NGT had similar difficulties judging the size of the available space and would sometimes have the hands (almost) touching although the objects they represented further apart, or the hands would run out of signing space (e.g., colliding with the torso). Again, the problem may be both cognitive and motoric, requiring the correct estimation of how much space is needed for a given representation and how the two hands need to be positioned toward each other in order to complete their movement unimpeded.

In summary, we see clear parallels between language acquisition and language emergence: Homesigners and young children acquiring an established signed language tend to omit either Figure or Ground in their depictions of motion events. Some adult M2L2 learners likewise omit one classifier in a CC. For children learning an established signed language such as TİD and HKSL, the evidence suggests that Ground is omitted more frequently than Figure. When they do represent both elements, they are typically encoded by separate signs at earlier stages, and simultaneously at later stages, after age seven (e.g., Sümer, 2015). Here, too, M2L2 learners sometimes choose sequential expressions. For ZFHS, the signing of Jane—the eldest signer—can be taken as representing an earlier stage in the emergence of the language; she tends to encode Ground and Figure using separate CCs. The signing of Will—the third-born deaf signer who acquired ZFHS from his older siblings—can be taken as representing a later stage of emergence. His encoding of Figure and Ground shows simultaneity in its linguistic organization.

### Path and Manner

Two additional properties of motion events are Path of motion and Manner of motion. Path refers to the trajectory along which the Figure moves (e.g., upward, downward, linear, circular, zig-zag shaped). Manner refers to the quality of the movement and is constrained by the characteristics of the moving entity (e.g., a ball may roll or bounce along a given path, while animate entities propel themselves in different ways, whether swimming, flying, running, jumping, etc.). In spoken languages, Path and Manner are typically expressed in separate lexical items (Talmy, 1985, 1991). For instance, in a “path-framed” language such as Spanish, Path is typically expressed in the main verb, while Manner is optionally expressed via a gerund or prepositional phrase, as in *la botella entró a la cueva flotando*, literally, “the bottle entered the cave floating” (Talmy, 1991: 488). In a “satellite-framed” language such as English, Path is typically encoded in a prepositional phrase while the main verb encodes Manner, as in “the bottle floated into the cave” (Talmy, 1991: 488). Path-Manner complementarity in verbal roots may fit into a larger picture of manner-result complementarity, a tendency for verbal roots to encode either the manner of an action or its result (as entailed by a directed path), but not both (Beavers et al., 2010).

#### Established signed languages

Sign languages distinguish at least two types of Manner (Supalla, 1990): *Manner of locomotion* (e.g., ‘walk’, ‘fly’, or ‘swim’) and *Manner of motion along a path* (e.g., ‘roll’, ‘bounce’, or ‘spiral’). In contrast to spoken languages, these two types of Manner are encoded differently in signed languages: Manner of locomotion is typically (but not always) encoded separately from Path^7^, while Manner of motion along a path is almost always encoded simultaneously with Path (Supalla, 1982, 1990). For instance, to represent a person running up a hill in ASL, signers will first use a body classifier to represent the motion of the arms and hands while running, followed by a “person” classifier handshape (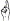) moving upward (Manner of locomotion + Path). In contrast, to show a vehicle spiraling along a downward path in ASL, one would move the “vehicle” classifier handshape (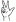) in a circular fashion while simultaneously moving it downward (Supalla, 1990: 129−133).

Path and Manner of locomotion are also sometimes expressed simultaneously within a single sign. Such signs may either be one- or two-handed: To represent a person walking (Manner of locomotion) upward (Path), a signer may wiggle the index and middle fingers of the upside-down V-hand (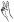) while moving the entire hand upward. Taub and Galvan (2001) provide an example of a two-handed Manner of locomotion + Path expression in ASL, in which a person shuffling (Manner of locomotion) sideways along a window ledge (Path) can be represented by the two index fingers moving sideways in a slow and careful manner.

Most cases of simultaneous Manner of locomotion + Path encoding involve the upside-down V (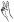 or “legs”) classifier. In contrast to body-part classifiers, this classifier does not trigger the simultaneous use of constructed action. It shows Manner via the movement of index and middle fingers (walking, jumping, propelling the body forward in water) and it shows Path by displacement of the entire hand through space^8^. When looking at the acquisition of Manner + Path predicates, we will thus focus on Manners that can be expressed with the 1-(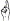) or V-(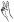) classifier, which allow for simultaneous encoding.

#### Acquisition of established signed languages

Newport (1981, 1988) looks at the acquisition of complex motion verbs involving Path and Manner components in ASL and finds that children start producing mostly target-like simultaneous constructions by ages four to five. Younger children either omit meaning components of the complex motion predicate or they produce a sequential string of Manner and Path predicates. All of Newport’s examples involve a straight or crooked -classifier (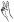 or 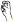, respectively) for humans or animals moving on legs. For example, she reports the depiction of a Fisher-Price man walking across the top of a roof. Adult ASL signers report the event with a complex motion predicate featuring a linear path movement combined with the V-classifier, which encodes simultaneous ‘walk’ Manner. In contrast, a child aged 4;5 produced a horizontal Path movement followed by a Manner verb for ‘walk’ without a path component. Younger signers may sometimes produce simultaneous structures, but do not do so consistently. For example, Slobin and Hoiting (1994) report on an ASL signer aged 3;8 who combines a walking Manner with a forward Path simultaneously.

Newport (1981) further reports two examples of a jumping or hopping Manner preceding a Path verb. In one case, a child (4;5) represented a hen jumping onto a barn roof with the crooked-V-classifier (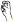) performing an arc-shaped jumping predicate followed by an upward Path predicate. In the second example, this same child described a cow hopping up a hill with the V-classifier hopping in place followed by a forward movement with her whole body to show Path. These examples demonstrate that sequentialization errors appear even in one-handed CCs. Thus, factors other than motor control issues can push children toward sequentialization.

Separating the Path and Manner representations of a single motion event results in a less iconic (or “analog,” in Newport’s terms) event representation, but it may reflect how children acquire not only CCs but language in general. Newport suggests that children’s perceptual and cognitive limitations (e.g., working memory limitations) lead to their perceiving and storing “excerpts” or components of complex constructions rather than the entire construction at once. For instance, a learner may perceive and store only the path of a complex movement (but not its manner) and therefore may store that path as a separate form. Selective perception and limited memory capacity may account for sequential productions of Path and Manner in younger children.

Singleton and Newport (2004) report on late learners of ASL exhibiting a similar tendency to encode each movement component via a separate sign. For instance, their late learners sometimes represented a car moving straight uphill as CAR MOVE STRAIGHT UPHILL, with separate signs for Motion, Path, and Direction. While children leave this analytical stage behind after roughly 5 years of ASL exposure, late learners may plateau in their acquisition, sometimes using CCs (e.g., WOMAN PASS DOG CL:1_*palm_down*_ + LINEAR ‘a woman passes by a dog,’ Singleton and Newport, 2004: 386) but sometimes using unanalyzed frozen forms.

#### Emergent signed languages

Few studies have examined the expression of Manner and Path in gesture. Özyürek et al. (2015) reported that hearing non-signers typically combine Manner and Path information holistically in a single gesture, no matter whether that gesture is concurrent with speech or not.

In the expression of motion, homesign systems represent an intermediate stage between gesture and full-blown language. Like (silent) gesturers, homesigners do not consistently segment Manner and Path (Özyürek et al., 2015). They can refer to Manner and Path individually, suggesting that they can at least isolate the two elements. For instance, homesigners represent Path trajectories by moving their hands through space, often using unmarked handshapes (e.g., an open palm 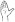 or the index finger 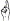) that do not provide information about physical characteristics of the Figure (Zheng and Goldin-Meadow, 2002). Homesigners also produce signs that represent Manner, but not Path: e.g., to represent the “fluttering” manner of falling snowflakes, one homesigner wiggled his fingers while keeping the hand at a single point in space (Zheng and Goldin-Meadow, 2002). However, homesigners do not typically concatenate Manner and Path gestures into larger strings as child learners of an established sign language do. In Özyürek et al.’s study, Turkish homesigners described roughly 50% of events with salient Manner and Path components via conflated forms in which Manner and Path were expressed simultaneously; most remaining events were described with only a Path component: ∼35%; or only a Manner component: ∼10%. In one example from Zheng and Goldin-Meadow (2002), a homesigner represented a frog hopping forward with an up-and-down motion of the elbow joint combined with a forward movement at the shoulder joint. Homesigners differ from gesturers in that they sometimes add an additional Manner or Path gesture in sequence with a conflated Manner + Path gesture, which Özyürek et al. (2015) argue represents an initial step toward language-like segmentation that only occurs when the gesture system is maintained over an extended period of time.

Turning now to emergent signed languages, we first discuss Nicaraguan Sign Language (NSL). This language emerged when deaf children were brought together at a newly established school for the deaf in the late 1970s (Senghas and Coppola, 2001). The children in this first cohort were likely homesigners before they arrived at the school. However, their homesigns quickly developed into a new language, NSL, which was adopted by subsequent cohorts of children who enrolled at the school. Presumably this happened in part because the homesigners were now members of a community centered around the school.

Senghas et al. (2004) examined the segmentation of Manner and Path in the co-speech gestures of hearing Nicaraguan Spanish speakers (which may have served as input for NSL signers) and three successive cohorts of signers of Nicaraguan Sign Language. For instance, to represent a cartoon character rolling down the hill, participants could conflate Manner and Path in a single sign (ROLL + DOWN; see Figure 1A in Senghas et al., 2004), or they could sequence them, producing a separate sign for each (ROLL DOWN; Figure 1B in Senghas et al., 2004). These authors found that the hearing Nicaraguans conflated Manner and Path in 100% of their gestured expressions of motion, and the first cohort of NSL signers did so in 75% of their motion expressions. However, in the second and third cohorts of NSL signers, Manner and Path were conflated in only 32% and 38% of expressions, respectively; in the majority of expressions produced by these later cohorts, Manner and Path were encoded in separate signs. Thus, there was a clear increase in segmentation as NSL was passed down through successive cohorts. Senghas et al. (2004) interpret these cross-cohort differences as a transition from a holistic, gesture-like stage to a more language-like stage characterized by discrete, linear structure. This re-structuring of the grammar of NSL likely reflects the learning mechanisms that children bring to the task of language acquisition. According to Senghas et al., these include predispositions for analytical structure and linear sequencing that drive children to break down “bundles” of information (such as the holistic gestures of the hearing Nicaraguans) into their constituent parts, and then re-combine those parts in sequence. This proposal is consistent with Newport’s proposal as to why children learning ASL produced errors in which Manner and Path were separated.

**FIGURE 5:**
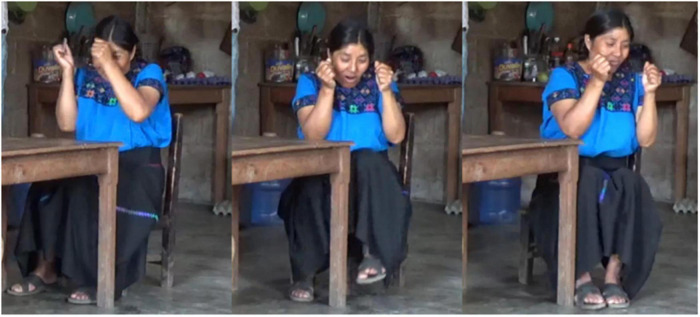
The first-born ZFHS signer represents Manner and Path simultaneously via constructed action.

**FIGURE 6:**
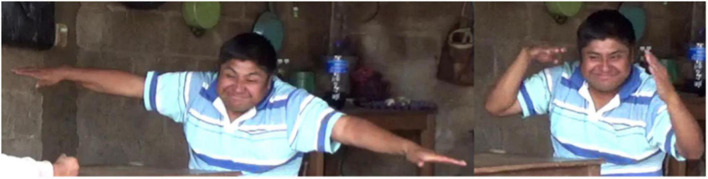
The third-born ZFHS signer represents Manner via constructed action, followed by Path via a classifier construction.

Parallel results have been obtained for Zinacantec Family Homesign (ZFHS) (German, 2022a). The first-born deaf ZFHS signer, who developed the original homesign system from scratch, with access only to gestural input, typically conflates Manner and Path. By contrast, all later-born signers, who received signed input from older signers, strongly prefer to sequence those elements. Furthermore, there is a shift from whole-body signing in the first-born signer to primarily manual signing in the later-born signers. Specifically, the first-born signer often adopts the perspective of the Figure and uses CA to enact the entire motion event. Thus, in order to encode Path she must move her body through space. For instance, in Figure 5, the first-born ZFHS signer describes a cartoon character walking while carrying a heavy object. She encodes Manner and Path by literally walking her feet out from under the table a short distance. By contrast, the later-born signers use CA only to encode Manner and encode Path through a manual CC, much as signers of established languages do. For instance, in Figure 6, the third deaf sibling describes a cartoon character flying into an enclosure. He begins by representing the Manner (‘flying’) via CA (outstretched arms), followed by a two-handed CC that represents the Path of the Figure into the Ground (i.e., the path of the cartoon character into the enclosure). The differences between the first- and later-born ZFHS signers indicate that even input provided by other homesigners is sufficient to scaffold the emergence of Manner/Path sequencing. The results for ZFHS thus parallel those of Senghas et al. (2004) for NSL, but extend them to a social group of a much smaller scale, indicating that regardless of the size of the signing community, emergent signed languages undergo a shift from holistic enactment toward sequential, combinatorial representations.

However, contrasting results have been obtained by Stoianov et al. (2022), who compare Cena, an emergent signed language of Brazil, with LIBRAS, the national sign language of Brazil. They elicited descriptions of motion events from 19 signers of each language using the Haifa clips, a set of video stimuli designed by Sandler et al. (2005). The authors found that signers of Cena and LIBRAS alike exhibited a strong preference for encoding Manner and Path of motion simultaneously. Thus, unlike the findings of Senghas et al. (2004) for NSL and those of German (2022a) for ZFHS, Stoianov et al. (2022) do not report a shift from simultaneous encoding to sequential encoding of Manner and Path as a signed language emerges. They argue that the shift from simultaneity to sequentiality is not universal among emergent languages, but is instead one of various possible outcomes depending on the sociolinguistic setting in which the language emerges. Specifically, they propose that a signed language that emerges when homesigners are brought together to form a signing community, such as NSL, will experience a shift from simultaneity to sequentiality, whereas a signed language arising in insular communities with a high rate of genetic deafness, such as Cena, will not. ZFHS fits neither of these profiles, yet seems to pattern like NSL in the encoding of Manner and Path. Further research is needed to determine the relationship between sociolinguistic setting and simultaneity in language emergence.

## Conclusion

In this paper, we have focused on simultaneity in two-handed expressions in signed languages. We have reviewed linguistic constraints on these expressions, discussed challenges that children and adult learners may face when acquiring them, and have synthesized the literature on the emergence of such expressions in young languages. Two hurdles that children may face in the acquisition of simultaneous expressions lie in the motor coordination of the two hands and in the cognitive load of representing many event components at the same time in an abstract space.

The literature on bimanual coordination in children suggests that they may struggle with inhibiting mirror movements in certain non-linguistic tasks requiring use of the non-dominant hand until ages eight to nine. In signed languages, Figure-Ground constructions and the use of discourse buoys require that the non-dominant hand be held in a particular configuration and in a particular location. This requires the suppression of any mirroring of the dominant hand. Logically, motor coordination difficulties could thus contribute to the late development of both structures.

Motoric complexity in children’s production of two-handed expressions is often reduced through the omission of information that would typically be encoded on the non-dominant hand. Thus, the Ground in Figure-Ground constructions and the usages of the non-dominant hand that maintain topics in narratives are often omitted. Even adults in TİD and DGS typically opt for non-simultaneous expressions of Ground and Figure. The timelines of children’s mastery of the inhibition of mirror movements and of signing children’s consistent inclusion of Ground line up: Both are mastered around age eight, suggesting that motor coordination may have some role in the late emergence of Figure-Ground constructions. However, we have too little direct evidence on how motoric complexity affects the development of two-handed sign forms, even of very young children’s acquisition of two-handed monomorphemic signs. A recommendation for future studies is this: independent measures of motor control skills in children would inform us as to whether motor control issues are indeed a limiting factor in children’s acquisition of two-handed constructions, including CCs and narratives.

Motor coordination is clearly not the only obstacle children have to overcome in producing simultaneous constructions. Even adult M2L2 learners, whose motor coordination skills are arguably more advanced than those of child learners, still struggle with encoding the many simultaneous components of a narrative via multiple articulators, and they sometimes omit classifiers in Figure-Ground CCs. Some constraints on children’s use of two-handed expressions seem to be independent of modality: for example, effective topic management across a discourse or narrative emerges around the same time in spoken and signed languages. In signed languages, weak-hand holds for topic maintenance and topic chaining start being used consistently around the same age as other narrative and discourse-structuring devices. More crucially, there are constraints on children’s use of simultaneously organized linguistic constructions even in expressions that are one-handed in signed languages. In acquiring one-handed CCs in which the language allows the simultaneous encoding of both the Manner and Path of a motion event, children separate them out before age five. They employ sequential encoding even at the expense of the iconicity that the visual-gestural modality allows. Moreover, our review has raised the possibility that the input with respect to two-handed Figure-Ground CCs might be less rich than we might have expected. Clearly, unexpected sequential constructions in children’s acquisition of signed languages are not just a response to the problems of coordinating linguistic expression across the two hands.

The cognitive load on the child likely plays a role here: Encoding many event components at the same time (whether they be the entities involved in the event or motion components such as Manner and Path) is cognitively demanding. De Weerdt (2020) argues that, even in adult signers, having two referents activated at the same time is demanding and therefore occurs more frequently if both referents are already known to the interlocutors. Children avoid layering of simultaneously occurring event components by either omitting some (e.g., the Ground in Figure-Ground constructions), or they express each component sequentially. In addition to tracking various event components, children also need to learn how to use the space in front of them as an abstract canvas onto which referents and their actions can be projected. Adult M2L2 learners, who struggle with the additional cognitive load of accessing a still developing language system, reduce the demands of encoding several event components simultaneously in similar ways as child learners: by omitting a classifier in a two-handed CC or by choosing a sequential expression.

When we compare children’s acquisition of simultaneous structures with how these structures develop in emerging sign languages, interesting parallels emerge, but there are also differences. With respect to Figure-Ground constructions, child learners tend to omit the Ground element while signers of emergent languages produce the two elements sequentially. In both cases, the result is an avoidance of simultaneity where it would be expected among adult signers of established languages (although recall that simultaneous expression of Figure and Ground appears to be less frequent in some established sign languages than we might have anticipated). With respect to Manner-Path constructions, the initial stages of acquisition and emergence differ, but their later stages are similar. While there is little data on whether child learners produce holistic forms initially, the earliest cohorts of signers of emergent languages rely primarily on holistic forms in which Manner and Path are produced simultaneously. In later stages of both acquisition and emergence (but see our earlier discussion of Cena), there is a tendency to produce Manner and Path sequentially. One explanation for this trajectory is that sequentiality and omission first arise when learners start breaking holistic signals up into their component parts. Children have to learn that CCs have sublexical structure; signers of emergent signed languages develop morphological structure by segmenting the linguistic expression of complex events into separate, sequential morphemes. Later, child learners start making full use of the potential of visual-gestural languages to layer information simultaneously and to thereby represent complex events iconically.

In language emergence, our review has revealed opposite patterns for Manner-Path (simultaneous then sequential) and Figure-Ground (sequential then simultaneous). This suggests that bimanual coordination could impact the emergence of simultaneously organized constructions. Representing Manner and Path simultaneously does not necessarily involve two hands, so—on this account—signers exploit simultaneity from the earliest stages of language emergence. By contrast, representing Figure and Ground simultaneously does indeed require that the signer coordinate the movements of the two hands. Thus, as in acquisition, motor coordination factors could form part of an explanation for why Figure and Ground are frequently expressed sequentially at the earliest stages of emergence. Much more research on the structure and acquisition of established signed languages and on the emergence of new signed languages is needed to understand the path toward simultaneity in visual-gestural languages.

## Ethics statement

Written permission from the copyright holder was obtained to reproduce the images in Figures 1, 2. AG’s research on ZFHS has been approved by the Institutional Review Board of the University of Texas at Austin, which waived the requirement for written informed consent based on the participants’ non-literacy and their cultural concerns about signing documents. Informed consent was obtained orally, including participants’ permission to publish their images (Figures 3–6) in research publications.

## Author contributions

CL wrote the parts of section “Introduction,” all of section “Acquisition of narrative and discourse functions of simultaneity,” and the first draft of section “Conclusion.” CL and AG wrote the section “Acquisition of classifier constructions.” AG wrote the subsections on emergent signed languages. RM wrote the section “Simultaneity in the lexicon: The two hands.” All authors contributed to the conception and structure of this manuscript and contributed to revisions of the manuscript, read drafts, and approved the submitted version.

## Footnotes

1 A range of names has been proposed for this class of constructions, among them *classifier verbs* or *predicates* (Supalla, 1990; Valli and Lucas, 1995), *depicting verbs* (Liddell, 2003), *poly-morphemic signs* (Engberg-Pedersen, 1993) or *poly-componential signs* (Schembri, 2003). We use the term *classifier construction* here because it is widely used but remain agnostic as to whether the term *classifier* is appropriate given the way that term has traditionally been used in the general linguistic literature.

2 Figures 1, 2 have been adapted from ASL-LEX (Caselli et al., 2017). We thank the creators of ASL-LEX for granting us permission to use these stills here.

3 The ASL sign SICK is an exception; the dominant hand contacts the forehead and the non-dominant contacts the torso.

4 Tkachman et al. (2021) report an analysis of dictionary data from ASL, British Sign Language (BSL), and Hong Kong Sign Language (HKSL); they found that two-handed symmetrical signs with alternating (out-of-phase) movement are typically repeated (unlike two-handed signs in which the two hands move in phase). They ascribe this result to central pattern generators involved in locomotion.

5 CCs may also encode how an object is handled or may give information about its visual characteristics, such as shape or size, but as most of the acquisition literature focuses on locative expressions, we will leave these aside for now.

6 Schick (1990b) reports that classifier handshapes are used correctly by age 4;5, while children as old as six to seven still have difficulty integrating spatial mapping into CCs.

7 The reason for sequential encoding seems to lie in the form of manner of locomotion predicates. Most classifiers used to describe how humans or animals move are body-part classifiers, e.g., index fingers () or B-hands () representing legs, feet, or paws, or B-hands () representing a swimmer’s hands or a bird’s wings. Body-part classifiers involve constructed action, whereby the signer’s body comes to represent the body of the moving entity. Thus, to encode Path simultaneously, a signer would have to move her entire body along the path trajectory. This strategy is unlikely to be employed since the lower extremities are not typically considered to be phonologically significant in established signed languages, although it is attested in performative registers of signing (Quinto-Pozos and Mehta, 2010). Subsequently, Supalla’s findings on sequential Manner of locomotion and Path encoding in ASL have been replicated for a number of signed languages: NGT (Slobin and Hoiting, 1994), Adamorobe SL (Nyst, 2007), Hong Kong SL (Tang and Yang, 2007), Argentinian SL, and Catalan SL (both Benedicto et al., 2008).

8 For a morphosyntactic analysis of the bi-eventive structure of the V-classifier in Russian SL, see Kimmelman et al. (2020). Tang and Yang (2007) note that the expressive potential of this classifier is somewhat limited, as it cannot represent manners of motion that saliently involve the hands. To represent ‘marching’ in HKSL, for instance, a body-part classifier predicate with arm movement has to precede the V-classifier showing Path and a ‘marching’ leg motion. Likewise, Benedicto et al. (2008) show an example from Argentinean SL of a horizontally oriented V-classifier following a body-part classifier for swimming. These examples show that signers add additional CCs in sequence because the V-classifier does not represent all the limbs involved in the manner of locomotion.
